# FOXO1-suppressed miR-424 regulates the proliferation and osteogenic differentiation of MSCs by targeting FGF2 under oxidative stress

**DOI:** 10.1038/srep42331

**Published:** 2017-02-10

**Authors:** Liangping Li, Qihua Qi, Jiaquan Luo, Sheng Huang, Zemin Ling, Manman Gao, Zhiyu Zhou, Maik Stiehler, Xuenong Zou

**Affiliations:** 1 Guangdong Provincial Key Laboratory of Orthopaedics and Traumatology, Orthopaedic Research Institute /Department of Spinal Surgery, The First Affiliated Hospital of Sun Yat-sen University, Guangzhou, 510080, P R China; 2 Centre for Translational Bone, Joint and Soft Tissue Research, Medical Faculty and University Centre for Orthopaedics and Trauma Surgery, University Hospital Carl Gustav Carus at Technische Universität Dresden, Dresden, 01307, Germany

## Abstract

Recently, microRNAs (miRNAs) have been identified as key regulators of the proliferation and differentiation of mesenchymal stem cells (MSCs). Our previous *in vivo* study and other *in vitro* studies using miRNA microarrays suggest that miR-424 is involved in the regulation of bone formation. However, the role and mechanism of miR-424 in bone formation still remain unknown. Here, we identified that the downregulation of miR-424 mediates bone formation under oxidative stress, and we explored its underlying mechanism. Our results showed that miR-424 was significantly downregulated in an anterior lumbar interbody fusion model of pigs and in a cell model of oxidative stress induced by H_2_O_2_. The overexpression of miR-424 inhibited proliferation and osteogenic differentiation shown by a decrease in alkaline phosphatase (ALP) activity, mineralization and osteogenic markers, including RUNX2 and ALP, whereas the knockdown of miR-424 led to the opposite results. Moreover, miR-424 exerts its effects by targeting FGF2. Furthermore, we found that FOXO1 suppressed miR-424 expression and bound to its promoter region. FOXO1 enhanced proliferation and osteogenic differentiation in part through the miR-424/FGF2 pathway. These results indicated that FOXO1-suppressed miR-424 regulates both the proliferation and osteogenic differentiation of MSCs via targeting FGF2, suggesting that miR-424 might be a potential novel therapeutic strategy for promoting bone formation.

Although bone repair materials have developed rapidly and are increasingly used, the development of strategies to promote bone formation and avoid fibrosis remains a big challenge in the field of bone and dental implantology. Bone formation includes the recruitment, commitment, proliferation and osteogenic differentiation of mesenchymal stem cells (MSCs)^1^. MSCs around bone repair materials undergo oxidative stress and a series of adaptive responses to maintain redox homeostasis to survive, proliferate and differentiate towards osteoblasts after implantation^2^. Bone formation after the implantation of bone repair materials is a more complex process that is highly controlled by growth factors, hormones and extracellular matrix and is influenced by the inflammation response, vascularization and oxidative stress^3^. Currently, the mechanism of bone formation after implantation is still not entirely understood.

MicroRNAs (miRNAs) are a class of small non-coding RNAs that play a key role in cellular processes, such as proliferation and differentiation, through post-transcriptional regulation^4^. Over the past few years, reports on the regulation of bone formation by miRNAs have emerged and continued to grow^5^. It has been reported that miR-424 is involved in the proliferation and osteogenic differentiation of MSCs using miRNA microarrays^6^,^7^, while the specific roles and mechanisms in these processes have not been investigated. In addition, emerging evidence demonstrates that miR-424 is downregulated by oxidative stress in patients and mice after ischaemic stroke^8^,^9^. Based on these observations, we hypothesized that the downregulation of miR-424 may be involved in proliferation and osteogenic differentiation under oxidative stress after implantation. Furthermore, the upstream and downstream regulatory mechanisms were investigated.

To defend themselves against oxidative stress, cells possess several redox defence systems, including antioxidant enzymes governed by the forkhead box O (FOXO) family that is involved in diverse cellular functions, such as proliferation and differentiation^10^. Among the FOXO family, FOXO1 and FOXO3 are the most important factors and have broad and overlapping functions^11^. Recent studies have shown that FOXOs are essential modulators of the proliferation and differentiation of osteoblastic precursors under oxidative stress^12^,^13^. On the other hand, fibroblast growth factor-2 (FGF2), a crucial member of a large family of proteins that regulate a wide range of cellular functions, is upregulated under oxidative stress^14^,^15^,^16^. Moreover, FGF2 can enhance the proliferation and osteogenic differentiation of MSCs to promote bone formation^17^,^18^. Although miR-424, FOXOs and FGF2 are all associated with bone formation, whether there is a regulatory relationship among these factors during bone formation under oxidative stress has not been defined.

Our previous studies have demonstrated that genes and miRNAs play important roles in bone formation after implantation of bone repair materials in the anterior lumbar interbody fusion (ALIF) model of pigs^3^,^19^. Our present work aimed at elucidating the effect of miR-424 on bone formation and the upstream and downstream regulatory mechanisms under oxidative stress. We identified that miR-424, which is repressed transcriptionally by FOXO1, regulates the proliferation and osteogenic differentiation of MSCs by targeting FGF2 under oxidative stress. Moreover, we found a novel mechanism for FOXO1 in which it stimulates the proliferation and osteogenic differentiation of MSCs through the miR-424/FGF2 pathway. Our results provide insight into the mechanism of bone formation after implantation and the relationship of FOXO1, miR-424, and FGF2 under oxidative stress.

## Results

### Establishment of a cellular model of oxidative stress

**Figure 1:**
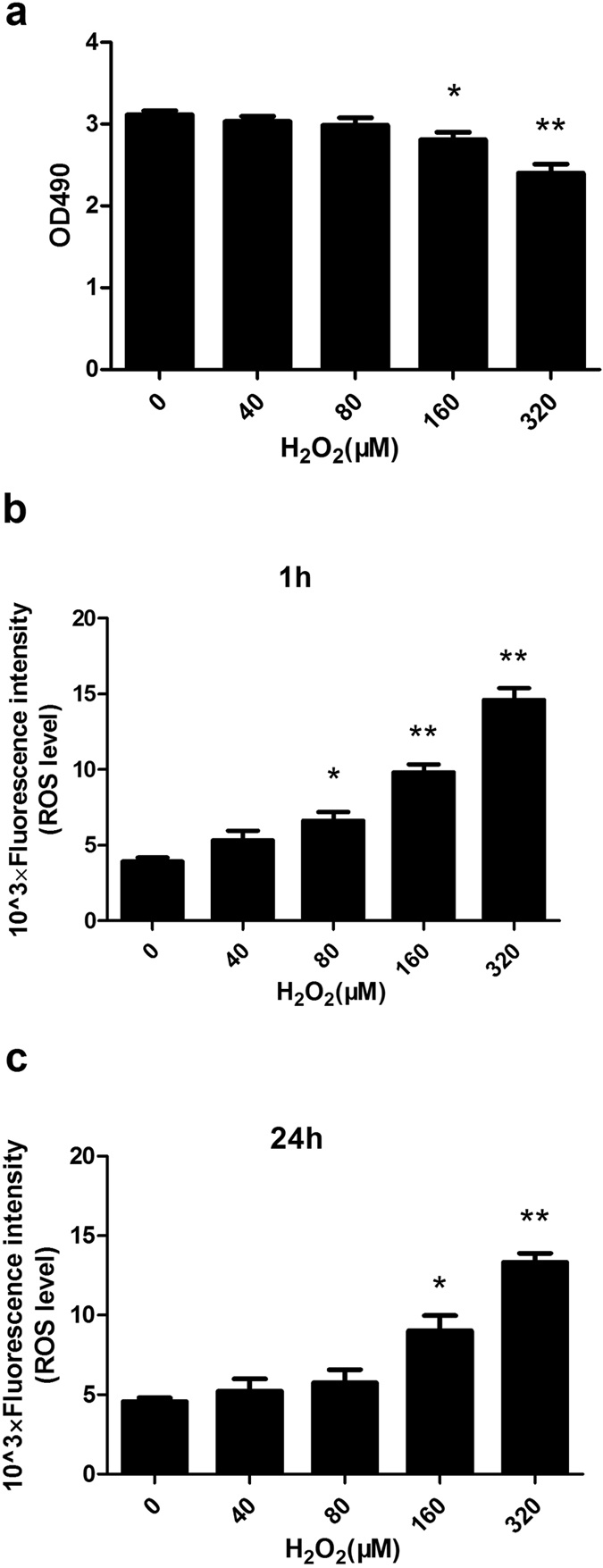
Cell viability and the levels of intracellular ROS of hASCs exposed to H_2_O_2_. (**a**) Cell viability was assessed with a MTS assay after hASCs were incubated with 40, 80, 160 and 320 μM H_2_O_2_ for 24 h; 0 μM H_2_O_2_ was used as the control group. (**b**,**c**) The levels of intracellular ROS at 1 h and 24 h were determined after hASCs were incubated with H_2_O_2_ at the above concentrations. The production of ROS was quantified by the amount of cellular DCF synthesis. The fluorescence intensity was measured using flow cytometry. *P < 0.05, **P < 0.01 compared to the control group. The results indicate the mean ± SD of triplicate experiments.

To establish a cell model mimicking oxidative stress conditions and redox homeostasis after implantation of bone repair materials, cell viability was first analysed. hTERT-transduced human adipose derived mesenchymal stem cells (hASCs) were treated with different concentrations of hydrogen peroxide (H_2_O_2_) for 24 h. H_2_O_2_ treatment resulted in decreased cell viability in a dose-dependent manner. Cell viability did not significantly decrease when hASCs were incubated with H_2_O_2_ at concentrations up to 80 μM (Fig. 1a).

To evaluate the status of oxidative stress at different stages in hASCs treated with H_2_O_2_, intracellular ROS was measured by flow cytometry. H_2_O_2_ treatment resulted in increased levels of intracellular ROS at both 1 h and 24 h in a dose-dependent manner. Compared with the control group, the levels of intracellular ROS at 1 h significantly increased when hASCs were exposed to 80 μM H_2_O_2_ (Fig. 1b). However, at 24 h, no significant increase was observed (Fig. 1c). These results suggest that hASCs initially undergo oxidative stress and subsequently maintain redox homeostasis upon exposure to 80 μM H_2_O_2_. Thus, hASCs incubated with 80 μM H_2_O_2_ were used as a cell model to mimic oxidative stress after implantation in the following study.

### Expression of miR-424, FOXOs, and FGF2 under oxidative stress

**Figure 2:**
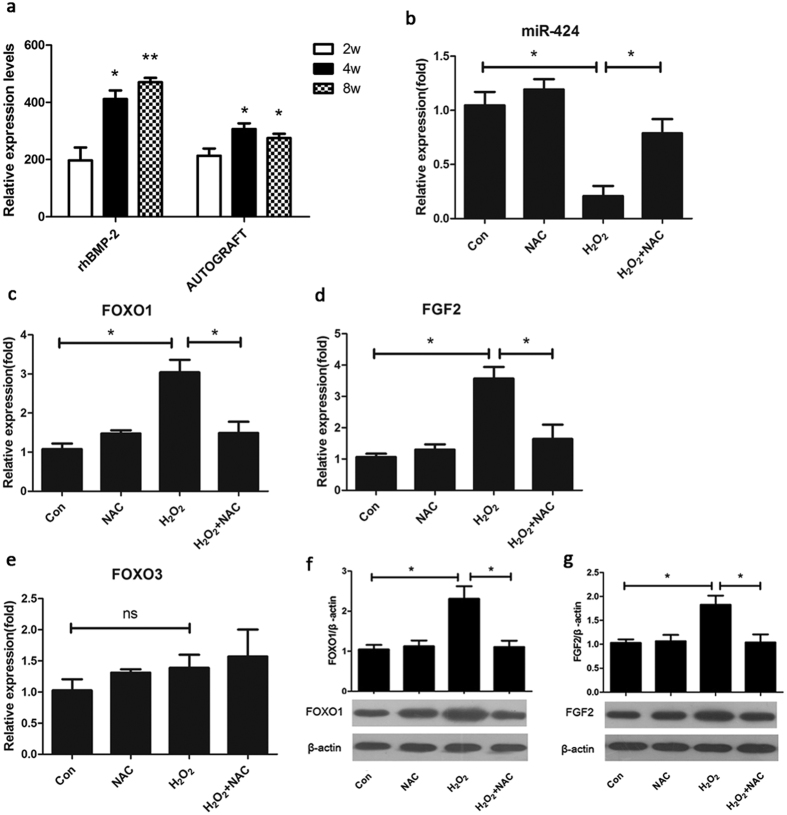
The expression of miR-424, FOXOs, and FGF2 under oxidative stress. (**a**) Relative expression levels of miR-424 expression after implantation of rhBMP-2 and AUTOGRAFT in the ALIF model of pigs at different time points. rhBMP-2, recombinant human bone morphogenetic protein-2/absorbable collagen sponge; AUTOGRAFT, autograft bone; ALIF, anterior lumbar interbody fusion. *P < 0.05, **P < 0.01 compared to 2 W, n = 3. Cells were exposed to 80 μM H_2_O_2_ for 24 h with or without pretreatment with 1 mM NAC; 0 μM H_2_O_2_ was used as the control group. The mRNA expression levels of miR-424 (**b**), FOXO1 (**c**), FGF2 (**d**) and FOXO3 (**e**) were determined by qRT-PCR. The protein expression levels of FOXO1 (**f**) and FGF2 (**g**) were determined by Western blot. *P < 0.05, ns, not significant. The results indicate the mean ± SD of triplicate experiments.

Our previous study demonstrated that miRNAs play important roles in bone formation by analysing the miRNA profiles in a porcine ALIF model after implantation of bone repair materials^19^. We found decreased miR-424 expression at 2 weeks compared to 4 and 8 weeks after implantation of rhBMP-2 and bone autograft in the ALIF model (Fig. 2a), suggesting that miR-424 expression decreased at an early stage of implantation when oxidative stress was at its peak. To further confirm the expression of miR-424 under oxidative stress, real-time quantitative polymerase chain reaction (qRT-PCR) was performed after hASCs were incubated with 80 μM H_2_O_2_ for 24 h. Compared with the control group, miR-424 was downregulated in hASCs exposed to H_2_O_2_. In addition, the antioxidant N-acetyl L-cysteine (1 mM, NAC) attenuated the inhibitory effect (Fig. 2b).

Moreover, we measured the expression of FOXO1, FOXO3 and FGF2 in the cell model of oxidative stress. Conversely, we found that the mRNA expression of FOXO1 and FGF2 was upregulated upon H_2_O_2_ treatment for 24 h, and NAC reduced the stimulatory effect as measured using qRT-PCR (Fig. 2c,d). The increase in the level of FOXO3 mRNA was insignificant (Fig. 2e). In addition, we confirmed that the protein expression of FOXO1 and FGF2 was upregulated upon H_2_O_2_ treatment for 24 h (Fig. 2f,g). Collectively, these findings indicate that miR-424, FOXO1, and FGF2 were simultaneously regulated by oxidative stress and that FOXO1 may be involved in maintaining redox homeostasis.

### MiR-424 inhibits the proliferation and osteogenic differentiation of hASCs under oxidative stress

**Figure 3:**
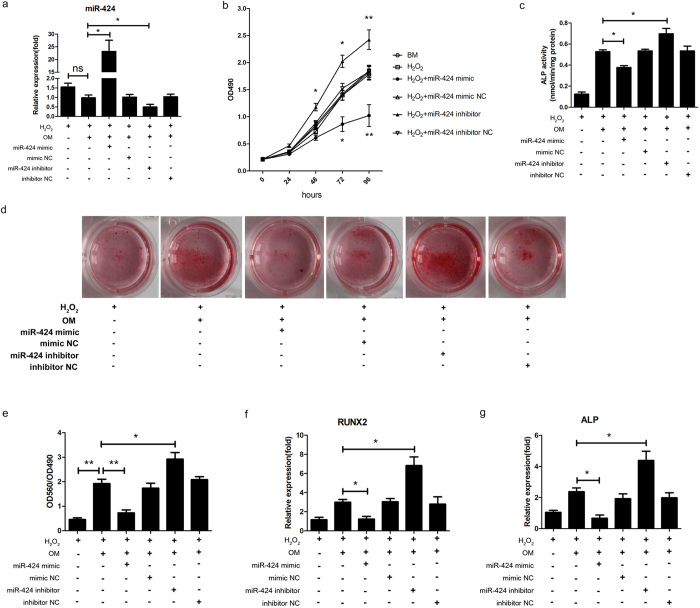
MiR-424 inhibits the proliferation and osteogenic differentiation of hASCs. hASCs were transfected with a miR-424 mimic and a miR-424 inhibitor for 24 h. A mimic NC (negative control) and an inhibitor NC were used as a negative control. Thereafter, the cells were incubated with basal medium (BM) or osteogenic medium (OM) and were continuously exposed to H_2_O_2_ until the indicated times. (**a**) qRT-PCR was performed to evaluate the expression of miR-424 at 3 days in response to the transfection of a miR-424 mimic and a miR-424 inhibitor. (**b**) The proliferation of hASCs after miR-424 overexpression and miR-424 knockdown was evaluated by a MTS assay. (**c**) ALP activity was evaluated after osteogenic induction for 7 days. (**d**) The mineralization of the cells was evaluated using Alizarin Red staining after osteogenic induction for 21 days. (**e**,**f**) The expression of RUNX2 and ALP, two key osteogenic markers, were evaluated after osteogenic induction for 7 days using qRT-PCR. *P < 0.05, **P < 0.01, ns, not significant. The results indicate the mean ± SD of triplicate experiments. The images shown are representative of three independent experiments.

To determine the roles of miR-424 in hASCs under oxidative stress, cells were transfected with a miR-424 mimic and a miR-424 inhibitor. At first, the transfection of the miR-424 mimic led to increased miR-424 expression at 3 days, whereas the transfection of the miR-424 inhibitor led to decreased miR-424 expression at 3 days (Fig. 3a). In addition, miR-424 expression was slightly downregulated during osteogenic differentiation, but it was not statistically significant (Fig. 3a). To determine the effect of miR-424 on the proliferation of MSCs, a MTS assay was performed after transfection. The overexpression of miR-424 significantly reduced the proliferation of hASCs in response to H_2_O_2_. Simultaneously, the inhibition of miR-424 significantly promoted the proliferation of hASCs (Fig. 3b).

Likewise, we investigated the effect of miR-424 on osteoblast differentiation in hASCs. After hASCs were incubated with osteogenic medium and continuously exposed to H_2_O_2_ for 7 days, alkaline phosphatase (ALP) activity was assessed. The ALP activity was significantly reduced in response to miR-424 overexpression and elevated in response to miR-424 knockdown (Fig. 3c). Moreover, we evaluated the effect of miR-424 on the mineralization of hASCs using Alizarin Red staining after osteogenic induction and continuous exposure to H_2_O_2_ for 21 days. The overexpression of miR-424 led to a significant decrease, whereas the inhibition of miR-424 led to a significant increase in the mineralization of hASCs (Fig. 3d,e). To further confirm these observations, we employed qRT-PCR to evaluate osteogenic marker genes. We found that the mRNA expression of RUNX2 and ALP, two key osteogenesis markers, changed in a manner consistent with the effects of miR-424 on the ALP activity and mineralization (Fig. 3f,g). Taken together, our results suggest that miR-424 inhibits the proliferation and osteogenic differentiation of hASCs under oxidative stress.

### MiR-424 targets FGF2, not FOXO1, under oxidative stress

**Figure 4:**
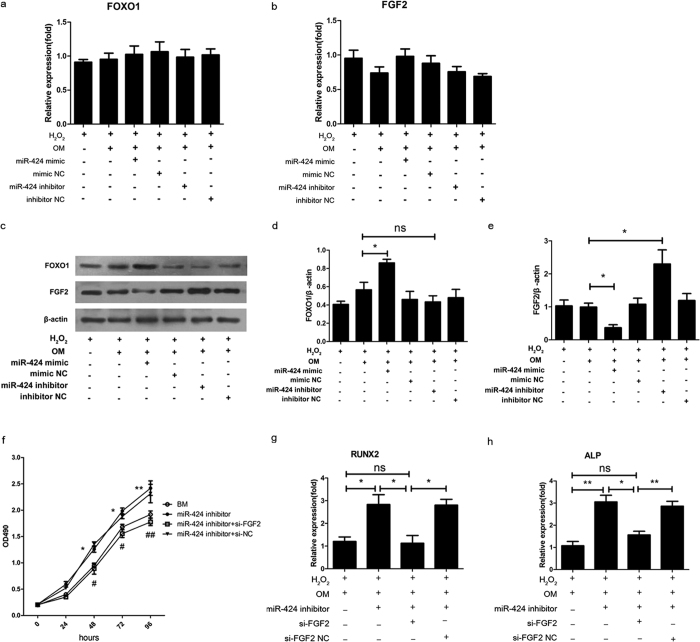
MiR-424 targets FGF2, not FOXO1. hASCs were transfected with a miR-424 mimic and a miR-424 inhibitor for 24 h. A mimic NC (negative control) and an inhibitor NC were used as a negative control. Thereafter, the cells were incubated with basal medium (BM) or osteogenic medium (OM) and were continuously exposed to H_2_O_2_ until the indicated times. (**a**,**b**) qRT-PCR shows the mRNA levels of both FOXO1 and FGF2 at 3 days in response to miR-424 overexpression and knockdown. (**c**,**d**,**e**) Western blot was performed to evaluate the protein expression of FOXO1 and FGF2 at 3 days in response to miR-424 overexpression and knockdown. (**f**) The proliferation of hASCs after cotransfection of a miR-424 inhibitor and FGF2 siRNA was evaluated using a MTS assay. (**g**,**h**) The mRNA expression levels of RUNX2 and ALP at 7 days after the cotransfection of a miR-424 inhibitor and FGF2 siRNA were evaluated by qRT-PCR. *P < 0.05, **P < 0.01, ^#^*P* < 0.05, ^##^*P* < 0.01 compared to the miR-424 inhibitor + si-NC group, ns, not significant. The results indicate the mean ± SD of triplicate experiments. The images shown are representative of three independent experiments.

To understand the underlying mechanism, we searched for potential targets for miR-424 in Targetscan, PicTar and miRBase using miRNA target prediction algorithms. Interestingly, among the predicted targets, we identified FOXO1 and FGF2 as candidates. These factors have crucial roles in bone formation and their expression have an inverse correlation with miR-424 expression under oxidative stress. We tested whether the upregulation of FOXO1 and FGF2 resulted from the downregulation of miR-424 under oxidative stress. The mRNA levels of both FOXO1 and FGF2 at 3 days were not significantly altered by the overexpression or inhibition of miR-424 in hASCs exposed to H_2_O_2_ (Fig. 4a,b). However, the protein expression of FGF2 at 3 days was significantly decreased by treatment with the miR-424 mimic and increased by treatment with the miR-424 inhibitor based on Western blot analysis (Fig. 4c,e). The protein expression of FOXO1 at 3 days increased in response to miR-424 overexpression, but it was not significantly altered by the inhibition of miR-424 (Fig. 4c,d). In view of the negative effect of miRNAs, our data suggest that miR-424 might target FGF2, not FOXO1, under oxidative stress.

Furthermore, we examined whether the knockdown of FGF2 reversed the proliferative and osteogenic effects of miR-424 downregulation on hASCs under oxidative stress. Indeed, the proliferative effect of miR-424 downregulation was abolished by FGF2 knockdown (Fig. 4f). Similarly, the positive effect of miR-424 downregulation on osteogenic differentiation was abrogated by FGF2 knockdown, as shown by the significantly attenuated mRNA expression levels of RUNX2 and ALP by FGF2 siRNA, even during treatment with the miR-424 inhibitor (Fig. 4g,h). Because it has previously been reported that miR-424 targets FGF2, the corresponding validation using a dual-luciferase reporter assay was not performed in our study. Collectively, these data indicate that miR-424 inhibits the proliferation and osteogenic differentiation of hASCs by targeting FGF2 under oxidative stress.

### FOXO1 negatively regulates miR-424 in a transcriptional manner

**Figure 5:**
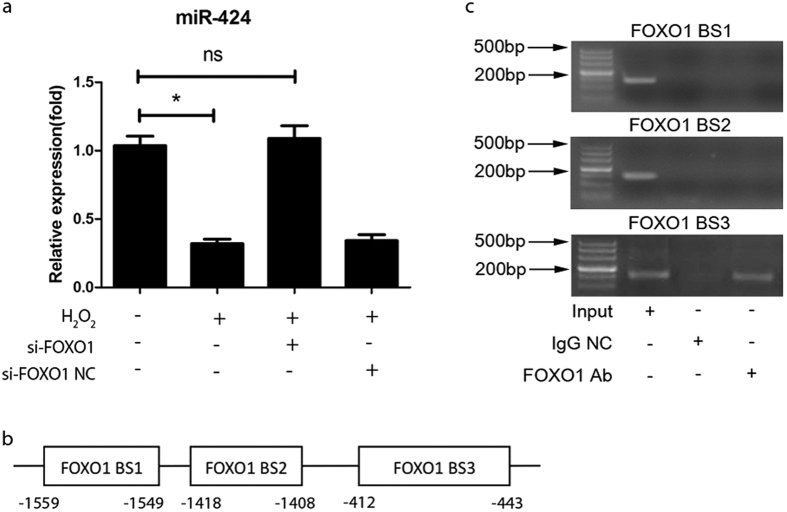
MiR-424 is repressed transcriptionally by FOXO1. (**a**) hASCs were transfected with FOXO1 siRNA for 24 h. siRNA NC (negative control) was used as a negative control. qRT-PCR showed that the knockdown of FOXO1 restored miR-424 expression in hASCs exposed to H_2_O_2_ for 24 h. *P < 0.05, ns, not significant. The results indicate the mean ± SD of triplicate experiments. (**b**) Schematic diagram of the three putative FOXO1 binding sites identified in the miR-424 promoter. (**c**) A ChIP assay shows that FOXO1 binds to the promoter of miR-424. Three optimal FOXO1 consensus binding sites (BS) in the promoter of miR-424 were identified according to the JASPAR database. Accordingly, we designed three primers containing FOXO1 BS1, BS2 or BS3. Chromatin-bound DNA was immunoprecipitated with the anti-FOXO1 antibody (FOXO1 Ab). IgG was used as a negative control. Thereafter, PCR was performed for the analysis of FOXO1 binding to the promoter of miR-424. The images shown are representative of three independent experiments.

As a key transcription factor in redox homeostasis, FOXO1 can transcriptionally regulate gene expression. Accordingly, we tested whether miR-424 expression is regulated by FOXO1. Indeed, the knockdown of FOXO1 using siRNA completely abolished the reduction in the level of miR-424 in hASCs exposed to H_2_O_2_ for 24 h (Fig. 5a). Furthermore, we tested whether it occurs in a transcription-dependent manner. Three optimal FOXO1 consensus binding sites (BS) in the promoter of miR-424 were identified according to bioinformatics analysis (Fig. 5b). Thereafter, we designed three primers containing FOXO1 BS1, BS2 or BS3 and performed PCR to determine whether FOXO1 binds to the promoter of miR-424 (Supplementary Table 1). A ChIP assay showed an increase in the association levels of FOXO1 with BS3. However, an association of FOXO1 with BS1 or BS2 was not detectable (Fig. 5c). These results suggest that miR-424 might be suppressed by FOXO1 in a transcriptional manner under oxidative stress.

### FOXO1 promotes proliferation and osteogenic differentiation through the miR-424/FGF2 pathway under oxidative stress

**Figure 6:**
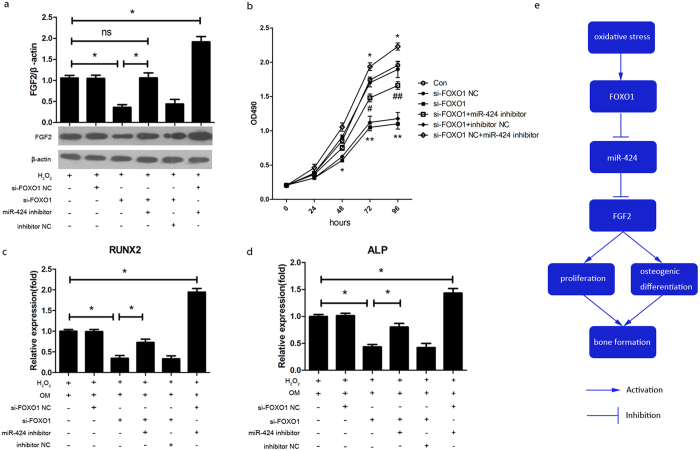
FOXO1 promotes proliferation and osteogenic differentiation through the miR-424/FGF2 pathway under oxidative stress. hASCs were transfected with a miR-424 mimic and a miR-424 inhibitor for 24 h. A mimic NC (negative control) and an inhibitor NC were used as a negative control. Thereafter, the cells were incubated with basal medium (BM) or osteogenic medium (OM) and were continuously exposed to H_2_O_2_ until the indicated times. (**a**) Western blot showed the protein expression of FGF2 at 3 days after the knockdown of FOXO1 and the cotransfection of FOXO1 siRNA or NC-siRNA and miR-424 inhibitor or inhibitor NC. (**b**) The proliferation of hASCs was evaluated by a MTS assay after the knockdown of FOXO1 and the cotransfection of FOXO1 siRNA or NC-siRNA and a miR-424 inhibitor or inhibitor NC. (**c**,**d**) The expression of RUNX2 and ALP were evaluated after the knockdown of FOXO1 and the cotransfection of FOXO1 siRNA or NC-siRNA and a miR-424 inhibitor or inhibitor NC and then osteogenic induction for 7 days. H_2_O_2_ + OM was used as the control group. *P < 0.05, **P < 0.01, ^#^*P* < 0.05, ^##^*P* < 0.01 compared to the si-FOXO1 + inhibitor NC group, ns, not significant. The results indicate the mean ± SD of triplicate experiments. The images shown are representative of three independent experiments. (**c**) A schematic representation of the main conclusions of this study.

To assess whether FOXO1 promotes proliferation and osteogenic differentiation through the miR-424/FGF2 pathway under oxidative stress, we first determined whether FGF2 is regulated by FOXO1 via miR-424 under oxidative stress. The transfection of FOXO1 siRNA and the cotransfection of FOXO1 siRNA and the miR-424 inhibitor led to decreased FOXO1 expression at 3 days (Supplementary Fig. 1). Then we found that the knockdown of FOXO1 led to the significantly decreased protein expression of FGF2 at 3 days in hASCs exposed to H_2_O_2_. In addition, the reduction was completely abrogated after the concomitant knockdown of FOXO1 and miR-424 (Fig. 6a). This result suggested that FGF2 expression is regulated by FOXO1 via miR-424.

Furthermore, the knockdown of FOXO1 led to the decreased proliferation of hASCs exposed to H_2_O_2_, whereas the knockdown of miR-424, in part, abrogated the anti-proliferative effect (Fig. 6b). In addition, during the osteoblastogenesis of hASCs, the knockdown of FOXO1 significantly reduced the mRNA expression of RUNX2 and ALP. However, the inhibition was rescued, in part, by the knockdown of miR-424 (Fig. 6c,d). Taken together, these data suggest that FOXO1 promotes proliferation and osteogenic differentiation, at least partly, through the miR-424/FGF2 pathway under oxidative stress.

## Discussion

Herein, we describe a novel miRNA-mediated regulation of bone formation *in vivo* and proliferation and osteogenic differentiation in a cell model of oxidative stress induced by H_2_O_2_, thus integrating these isolated observations into a cohesive mechanism. Our findings provide three major conclusions (Fig. 6e): (i) the downregulation of miR-424 facilitates proliferation and osteogenic differentiation via the upregulation of FGF2 in the context of oxidative stress, (ii) FOXO1 transcriptionally suppresses miR-424 expression, and (iii) FOXO1 enhances proliferation and osteogenic differentiation, at least partly, through the miR-424/ FGF2 pathway.

Oxidative stress involves excessive intracellular levels of reactive oxygen species (ROS) that result from an imbalance between the production and scavenging of ROS^20^. Our previous study demonstrated that the titanium alloy implants that are most frequently used in orthopaedics induce the production of ROS in cells^21^. Tsaryk *et al*. found that ROS are produced by human endothelial cells seeded on a titanium alloy upon H_2_O_2_ treatment and the cells possess the ability to maintain redox homeostasis to some extent^2^. Moreover, several animal studies have revealed that oxidative stress occurs at an early stage and then redox balance is gradually established during fracture healing. For instance, malondialdehyde, a marker of oxidative stress, was increased on days 7 and 14 and then decreased to basal levels after 28 days during fracture healing in rats^22^. Another study found that there was a temporary increase in the levels of the oxidative marker and that an 8-week period was sufficient to re-establish redox balance during bone healing in a rabbit model^23^. Our data revealed that the levels of intracellular ROS were significantly increased in hASCs exposed to 80 μM H_2_O_2_ for 1 h. In contrast, there was no significant increase after 24 h in the control group. Consistent with previous observations, our data suggest that the levels of intracellular ROS increased at an early stage and then decreased to normal levels because of redox homeostasis systems when hASCs were exposed to 80 μM H_2_O_2_. Thus, it is reasonable to consider that the cell model using 80 μM H_2_O_2_ can simulate the scenario of oxidative stress and redox balance after implantation.

MiR-424 is a cancer repressor involved in tumour cell proliferation, migration, and invasion^24^,^25^,^26^. It can modulate monoblastic cell differentiation^27^,^28^, vascular endothelial and smooth muscle cell phenotype and angiogenesis^29^. In addition, miR-424 is involved in the regulation of adipogenic differentiation of hASCs^30^. Moreover, the expression of miR-424 in human bone marrow–derived mesenchymal stem cells (hBMSCs) is higher than in osteoblasts^31^. Recently, decreased miR-424 expression was identified in osteogenically differentiated MSCs using miRNA microarrays^6^,^7^, suggesting that miR-424 expression is negatively correlated with osteogenic differentiation. In agreement with these observations, our present study elaborately demonstrates that miR-424 inhibits both the proliferation and osteogenic differentiation of hASCs, as shown by decreased OD490 values, ALP activity, calcium deposition and osteogenic markers, including RUNX2 and ALP, after the overexpression of miR-424 as well as the opposite effects after the inhibition of miR-424. Howerver, there exists a discrepancy in our study that although the expression of miR-424 was slightly downregulated, it did not significantly decrease during osteogenic differentiation.

Furthermore, the underlying molecular mechanism was investigated. Although both FGF2 and FOXO1 are predicted targets of miR-424, we found that miR-424 only negatively regulates FGF2, which is in turn known to be a regulator of various cell processes including proliferation and differentiation^32^. The observations that the protein expression of FGF2 was negatively regulated by miR-424 and that miR-424 has no effect on the mRNA expression of FGF2 indicate that FGF2 is regulated by miR-424 at the post-transcriptional level. Moreover, we found that the inhibition of FGF2 using siRNA abolished the positive effects of miR-424 knockdown on the proliferation and osteogenic differentiation of hASCs exposed to H_2_O_2_, suggesting that miR-424 exerts its effects by targeting FGF2 under oxidative stress. Because miR-424 has been validated to target FGF2 in pulmonary artery endothelial cells using a luciferase report assay by Kim *et al*. in a recent study^33^, we speculate that miR-424 probably targets FGF2 by binding to the 3′ untranslated region (UTR) in hASCs as well, but we did not further perform the validation.

MiRNAs remain at a constant level under physiological conditions. Their levels, which are closely associated with their functions, change in response to the alteration of circumstances^34^. We found that miR-424 is significantly downregulated in the porcine spine fusion and in a cell model of oxidative stress induced by H_2_O_2_. Thus, it is important to explore the upstream molecular mechanism. Our finding that the knockdown of FOXO1 completely abolished the reduction of miR-424 in hASCs exposed to H_2_O_2_ suggested that FOXO1 mediates the downregulation of miR-424 under oxidative stress. Furthermore, a ChIP assay validated that FOXO1 can bind to the promoter region of miR-424. Previous reports have shown that miRNA expression can be regulated by transcriptional factors^34^,^35^. For instance, miR-21 is transcriptionally suppressed by FOXO3a^36^. Thus, it is suggested that the downregulation of miR-424 is mediated by FOXO1 in a transcriptional manner under oxidative stress.

FOXO1 belongs to the winged helix/forkhead family of transcription factors that is characterized by a 100-amino acid monomeric DNA-binding domain called the FOX domain. FOXO1 is highly expressed in the areas of intramembranous bone formation, such as the calvaria, and endochondral bone formation, such as the diaphysis of long bones^37^. Various studies have demonstrated that FOXO1 is required for redox balance in osteoblasts and skeletal homeostasis, which highlights the importance of the transcription factor in bone formation^38^,^39^. However, its specific roles in bone formation remain controversial. Some studies have shown that FOXO1 promotes bone formation by not only maintaining redox balance and protein synthesis^12^,^13^ but also enhancing the expression of RUNX2^37^,^40^. In contrast, other studies have demonstrated that FOXO1 attenuates bone formation by suppressing Wnt signaling by diverting β-catenin from TCF- to FOXO-mediated transcription^41^,^42^. Such a discrepancy raises the question as to whether there is any other mechanism by which FOXO1 regulates bone formation. Consistent with the former idea, our findings revealed that FOXO1 promotes the proliferation and osteogenic differentiation of hASCs under oxidative stress. Furthermore, we found a novel mechanism in that FOXO1 promotes bone formation, at least partly, through the miR-424/FGF2 pathway. Taken together, these observations indicate that FOXO1 exerts different roles in bone formation, depending on the type of cell and its stage of differentiation.

One of the limitations of the present study is that the effects and mechanisms of miR-424 on the proliferation and osteogenic differentiation of MSCs were mainly investigated *in vitro* without further verification in an animal model. Thus, we are cautious to extend the effects of miR-424 on *in vivo* bone formation and the underlying mechanism. We intend to further verify our findings using an animal model with implantation of bone repair materials in a future study. Another limitation is that we did not evaluate other validated and potentiated targets of miR-424. However, we found that the inhibition of FGF2 was sufficient to fully counter the effects of miR-424 knockdown on hASCs. In addition, the current study focused on the regulatory mechanism of miR-424 that links FOXO1 to FGF2 during bone formation. Nevertheless, we cannot completely eliminate the possibility that other validated targets of miR-424, such as cell-cycle checkpoint kinase 1 (Chk1)^24^, Cdc25^25^, c-Myb^43^, CTNNBIP1^26^, NFI-A^27^, and CDX2^44^, may also be involved in the proliferation or osteogenic differentiation of hASCs, which are also likely to mediate an increase in the protein expression of FOXO1 in response to miR-424 overexpression.

## Conclusion

In summary, the results obtained in this study revealed that FOXO1-suppressed miR-424 exhibited dual effects by controlling both the proliferation and osteogenic differentiation of MSCs by targeting FGF2 under oxidative stress. In addition, FOXO1 enhances bone formation, at least partly, through the miR-424/FGF2 pathway. These findings support the development of new strategies of inhibiting miR-424 and augmenting FOXO1 and FGF2 to guide the modification of bone repair biomaterials and promote bone formation.

## Materials and Methods

### Chemicals and reagents

All chemicals (H_2_O_2_, N-acetyl-L-cysteine (NAC), dexamethasone, ascorbic acid, β-glycerophosphate, 2′,7′-dichlorofluorescein diacetate (DCFH-DA), methylthiazolyltetrazolium (MTS), p-nitrophenylphosphate (pNPP), Alizarin Red S, cetylpyridinium chloride (CPC), and Triton X-100) were purchased from Sigma-Aldrich (St. Louis, MO, USA). The primary antibodies against FOXO1 and FGF2 were purchased from Abcam (Beverly, MA, USA).

### Cell culture and treatment

hTERT-transduced human adipose derived mesenchymal stem cells (hASCs) were purchased from the American Type Culture Collection (ATCC; Manassas, VA, USA). Cells were cultivated in human stem cell medium supplemented with 10% fetal bovine serum (basal medium, BM) at 37 °C in a humidified 5% CO_2_ atmosphere. The medium was replenished every three days.

H_2_O_2_ was used to induce oxidative stress after hASCs were seeded for 24 h. For osteogenic differentiation, fresh osteogenic medium (OM, 0.1 μM dexamethasone, 50 μg/ml ascorbic acid, and 10 mM β-glycerophosphate) was used for osteogenic induction after the cells reached 70–80% confluence.

### Cell viability and proliferation

Cell viability and proliferation were probed using a MTS assay. Cells were seeded in 96-well plates at 1 × 10^4^ cells/well. After cell culture for 24 h in an incubator, different doses of H_2_O_2_ were added. Cell viability was evaluated after the cells were treated with H_2_O_2_ for 24 h. Cell proliferation was evaluated after the cells grew for 0, 24, 48, 72, 96 h upon treatment with H_2_O_2_. The cells were rinsed with PBS once, and then, 120 μl of fresh medium containing 20 μl MTS was added to all test and control wells. After incubation at 37 °C for 3 h, the absorbance at 490 nm was measured using a spectrophotometric plate reader.

### Transfection of miRNAs and siRNA

A chemically synthesized miRNA mimic and inhibitor (Ribobio, Guangzhou, China) were used to augment and inhibit miR-424 function, respectively. Both the mimic (mimic NC (negative control)) and inhibitor (inhibitor NC) were used as a negative control. After seeding for 24 h, cells were transfected with 50 nM miR-424 mimic, mimic NC, 100 nM miR-424 inhibitor, and inhibitor NC or 50 nM FOXO1 siRNA, FGF2 siRNA, and NC siRNA for 24 h using the riboFECT™ CP Transfection Kit according to the manufacturer’s protocol (Ribobio, Guangzhou, China).

### ROS measurement

Intracellular ROS measurement was evaluated using a method previously described with slight modification^45^. Cells were seeded in 6-well plates at 2 × 10^5^ cells/well. After culturing for 24 h in the incubator, different doses of H_2_O_2_ were added. In brief, after the cells were exposed to H_2_O_2_ for 1 h or 24 h, the cells were incubated in growth medium containing 20 μM DCFH-DA for 30 min at 37 °C. After removal of the DCFH–DA-containing medium, the cells were rinsed with PBS to remove the residual extracellular DCFH-DA. The cells were detached with trypsin and resuspended after centrifugation. The fluorescence levels of the samples were measured using flow cytometry with the excitation and emission wavelengths set at 488 and 525 nm, respectively.

### Alkaline phosphatase (ALP) activity assay

To measure the ALP activity, cells were seeded in a 96-well plate and incubated with osteogenic medium containing H_2_O_2_. After osteogenic induction for 7 days, the cells were lysed with 1% Triton X-100 for 1 h at 4 °C. A portion of the cell lysate was incubated with pNPP in a buffer (0.1 M glycine, 1 mM MgCl_2_, and ZnCl_2_, pH 10.3) at 37 °C for 30 min. The reaction was quenched by adding 2 N NaOH, and the absorbance was measured at 405 nm. The resulting supernatant was used for the measurement of the protein concentration with a BCA protein assay kit. The ALP activity was expressed as nmol/min/mg of protein.

### Alizarin Red staining and quantitative detection

The mineralization of hASCs was probed using Alizarin Red staining. The cells were incubated with osteogenic medium and exposed to H_2_O_2_ after the transfection of the miRNAs. At 21 days, a portion of the samples was used to measure cell number by a MTS assay (OD490), and the remaining samples were used for staining. For Alizarin Red staining, cells were fixed with ice-cold 70% ethanol for 1 h. The cells were stained with 40 mM Alizarin Red S for 15 min at room temperature. After rinsing with distilled water to completely remove the unbound stain, the cells were visualized and imaged using a light microscope (Nikon, Eclipse TS100, Japan) and digital camera (Canon, EOS 700D, Japan). After drying, staining was eluted with 10% CPC in 10 mM sodium phosphate, pH 7.0, for 15 min and the absorbance was measured at 560 nm (OD560). To quantitatively compare the mineralization, the value was expressed as OD560/OD490.

### RNA isolation, cDNA synthesis and qRT-PCR

Total RNA was extracted using TRIzol reagent (Invitrogen, CA, USA) according to the manufacturer’s instructions. Total RNA (500 ng for mRNA or 1000 ng for miRNA) from each sample was subjected to reverse transcription using a commercially available kit according to the manufacturer’s protocol (TaKaRa, Japan). The primers used to amplify the target mRNA and miR-424 are listed in Table 1. All experiments were performed using a real-time PCR system (CFX ConnectTM Real-time system, Bio-Rad, USA). The expression levels of each mRNA and miR-424 were normalized to that of GAPDH and U6, respectively. The relative expression levels of all genes were calculated using the 2−ΔΔCt method.

### Western blot analysis

Cells were harvested for protein extraction using RIPA buffer supplemented with a protease inhibitor cocktail (Sigma-Aldrich). The total protein concentration was determined using a BCA protein assay kit according to the manufacturer’s instructions (Thermo Fisher Scientific, Rockford, IL, USA). Thirty micrograms of protein were separated by SDS-PAGE and then transferred onto a 0.45 μm PVDF membrane (ImmobilonTM, Millipore Corp., Bedford, MA). The membranes were blocked and then probed overnight with primary polyclonal anti-FOXO1, anti-FGF2 (1:500, Abcam) or β-actin (1:3000, Abcam) antibodies. The antibodies were detected using enhanced chemiluminescence with HRP-conjugated secondary antibodies (Jackson Immuno Research). The values of the band intensities were quantified using ImageJ software.

### Chromatin immunoprecipitation assay

Immunoprecipitation of FOXO1 with the miR-424 promoter was performed using the polyclonal mouse anti-FOXO1 antibody (ChIP grade; Abcam) and the Chromatin Immunoprecipitation Kit (EZ-ChIP™ Millipore). Briefly, the cells were incubated with 1% formaldehyde at room temperature for 10 min. The crosslinking was quenched with glycine for 5 min. The cells were lysed in a lysis buffer at 4 °C, and then, the lysates were sonicated into chromatin fragments with an average length of 500–800 bp as assessed by agarose gel electrophoresis. The samples were precleared with Protein A-agarose on a rocking platform at 4 °C. Then, 5 g of the specific antibodies was added and rocked at 4 °C for overnight. For the analysis of the binding of FOXO1 to the promoter of miR-424, PCR was performed with the primers that encompass FOXO1 BS1, BS2, BS3 of the human miR-424 promoter. The oligonucleotides were as follows: BS1 (−1559 bp binding site; forward, 5-CCTTTGACACCGATGCTGTT-3; reverse, 5-TCCCAACATTTTGTTCCACA-3); BS2 (−1418 bp binding site; forward, 5-TGTTGGGAGAAAGTTGTGGA-3; reverse, 5-CCATCTTGTTTTGGCAATGA-3); BS3 (−412–−443 bp binding site; forward, 5-AAGCTACCGGTGAGGTTTTG-3; reverse, 5-TGAAACTTCTTCCTGCTGCAT-3).

### Animal model and miRNA microarray experiment

The ALIF model and miRNA microarray experiment were elaborately described in our previous study^19^. Briefly, eighteen Danish female landrace pigs, 3-month-old, weighing approximately 50 kg, were used. Each was treated with a 3-level ALIF procedure at L3-4, L4-5 and L5-6 under general anaesthesia. The custom-made polyetheretherketone (PEEK) interbody cage with equine BPE (COLLOSS^®^ E; Ossacur AG, Oberstenfeld, Germany), rhBMP-2 dissolved on ACS (INFUSE^®^; Medtronic-Sofamor Danek, Memphis TN), or autograft bone were randomly inserted into each operation level. Each six pigs were terminated at each time-point of 2, 4, or 8 weeks. Total RNA samples were analyzed by CapitalBio Corporation (Beijing, China) for miRNA microarray experiments. MiRNA expression profiling was performed for each pooling RNA sample separately on the GeneChip^®^ miRNA 2.0 Array (Affymetrix) at CapitalBio Corporation.

### Statistical analysis

The data are presented as the mean ± SD. The difference between two groups was probed using Student’s *t*-test. The difference among multiple groups was evaluated using a one-way ANOVA followed by Bonferroni post-tests. Statistically significant results were indicated as *P < 0.05, **P < 0.01, ^#^*P* < 0.05, ^##^*P* < 0.01. Each experiment was repeated independently at least three times.

## Additional Information

**How to cite this article**: Li, L. *et al*. FOXO1-suppressed miR-424 regulates the proliferation and osteogenic differentiation of MSCs by targeting FGF2 under oxidative stress. *Sci. Rep.* **7**, 42331; doi: 10.1038/srep42331 (2017).

**Publisher's note:** Springer Nature remains neutral with regard to jurisdictional claims in published maps and institutional affiliations.

## Supplementary Material

Supplementary Information

## Figures and Tables

**Table 1:** Nucleotide sequences of the RT-PCR primers used.

Gene	Primer sequence
miR-424	5′-GCAGCAGCAATTCATGTTTTGAA-3′ (F)
U6	5′-CTCGCTTCGGCAGCACA-3′ (F)
	5′-AACGCTTCACGAATTTGCGT-3′ (R)
FOXO1	5′-GGCTGAGGGTTAGTGAGCAG-3′ (F)
	5′-AAAGGGAGTTGGTGAAAGACA-3′ (R)
FGF2	5′-AGCCAGGTAACGGTTAGCAC-3′ (F)
	5′-GGAGAAGAGCGACCCTCAC-3′ (R)
RUNX2	5′-ACTTCCTGTGCTCGGTGCT-3′ (F)
	5′-GACGGTTATGGTCAAGGTGAA-3′ (R)
ALP	5′-ACCATTCCCACGTCTTCACATTT-3′ (F)
	5′-AGAC ATTCTCTCGTTCACCGCC-3′ (R)
GAPDH	5′-TCGACAGTCAGCCGCATCTTCTTT-3′ (F)
	5′-GCCCAATACGACCAAATCCGTTGA-3′ (R)
