# Supplementary material for: FOXO1-suppressed miR-424 regulates the proliferation and osteogenic differentiation of MSCs by targeting FGF2 under oxidative stress
Source: Sci Rep. 2017 Feb 10;7:42331. doi: 10.1038/srep42331 (PMC5301230; doi:10.1038/srep42331)
Supplement: Supplementary Information [file srep42331-s1.pdf]

FOXO1-suppressed miR-424 regulates the proliferation and osteogenic differentiation of MSCs by targeting FGF2 under oxidative stress

Liangping Li [1,2+], Qihua Qi[1+], Jiaquan Luo[1], Sheng Huang[1], Zemin Ling[1], Manman Gao[1], Zhiyu Zhou[1], Maik Stiehler[2] & Xuenong Zou[1*]

[1]Guangdong Provincial Key Laboratory of Orthopaedics and Traumatology, Orthopaedic Research Institute /Department of Spinal Surgery, The First Affiliated Hospital of Sun Yat-sen University, Guangzhou, 510080, P R China

[2]Centre for Translational Bone, Joint and Soft Tissue Research, Medical Faculty and University Centre for Orthopaedics and Trauma Surgery, University Hospital Carl Gustav Carus at Technische Universität Dresden, Dresden, 01307, Germany

* Corresponding author: zxnong@hotmail.com (X. Zou)

[+]These authors contributed equally to this work.

>mir424 promoter

GAGGGGCGGGGAGAGAAAACTGAGTAAGGGGCAGTGACCGACTGTCAGCCTCGCTAGGGTCGGGAGCGACTG
GTTTTGACAGAATGTAAATGAACTTTTCCTCTTATTTGCAAGAAGTTAGTTATGTCTTGGAAAGAAATGTGT
GATTAATTTCACATTGTCTCTCCCGAATTAAAACTTTAAAGGTTGTTTAGTTTTCCCCGTAGGGACTTGACA
TTGTGGAAGTTTGGCTAGAATGCGCCGAGAGAAGGTACATCGTGTGTTTGGGGTGTGGGGGACGAAGGCATA
TGCAAAATTAAAAGACAAAACGTTGTAGAGTATGAACAGAGGAAGAGGCGTATTCTTTGGCTCTTAAATCAT
TTAGCCAAACACATTGCAGTTCAGAAGGGGGTGGGGCTATGGCAACTCGAAAGTATCGATTCTTTGTCTATT
GTATCAGTGAATGACCCTCTCCTGAAGTGACAAGACGCCCCTTTGTAATCTCTTTTCGTAAGTAAATTTCAG
TCTAAGGGGAAATATGTAAACTAGGGGATCGAGGTGGCTTTTTAGGGGTGTAACTCGTTGTACTATCACTGG
GAGAGATGAAAGGGGTTAGGGTGAAAGGTATAAATGAATTTGATTGTGGAACAAAATGTTGGGATGTGGAAC
AAAATGTTGGGATCCAAGAGCAATCTGGAAGGGACCTTCGCCTTTGACACCGATGCTGTTTCGAATGAACTT
TTGTTTTTCTAAGTAGTCAACTAAGGAAATGTTAAATTAGGCATGACTTTAACTTCTGACATGATGGCTCGT
ATATTTGTGTGGAACAAAATGTTGGGAGAAAGTTGTGGAGTTTTACATGATAATTGTACATTTGCTAAACAT
GTTTTTTTTTTCTTTTTAAGGGCAGTCAACGACATTTTTCTCCATTAATCCCAAAGTCAGTGTAAAAGTTGT
TTGTAAATATCATTGCCAAAACAAGATGGAATGTAGAGATACTTGATTGTCGCAATTTAGGGAATGGGGTTA
TTGTTACTAATGACTTTTTTTTTTTACCATGACATAGTGTATTTGTTACCACTGGATCTCAGTCTGGATGTT
ATAAATTCGATACTTAATCTTATTAAATACTCTGCACATTTTATTAACTAATAAATAGGACACTTAACATTC
ATTGTACTGGTTTTGAAAAACCGACTTAATTTGTAAGTGTAACAAGATACTTACAGTTGGATAATTTAAATG
TCTATTACTTGTGCTGAAATGTATTTAACTTTCAAAACCATTACAGTTAGTGCTGATTTTGTTGTTTTAACT
TTTAGGTTTGTGATTGAGGATTTCAGAGTGGGTTTTTTTTTAATGCTTAATTGAATATTGTATTCAAACAA
AAAGAGGGCATGCATGTGACTTGTTGCTAATATGTGTCCATTTTAAAAAGGAAAAAGTGGTATCTAATGAGT
TAATCATTAAGGAGTATGCGCAGTGCCGGCCATTTCAGGACATGAAAGTTTCTGTGGAGCTGCTTGAAACAG
GAAGGAGCGACTTGACATCAAGTATTTAAACTTTTTGATTTATGTTTTGATAGATGACATTGATAGGTATAA
ATTATAAATAAGTTTGAGTAACCAGTTTAATATGTAAACTGGTATTTCATCTACAGAAAGTTCTCTGTCATT
GTTTGGAGCTCTTTAAAATAACTCAGCTGGGGTGTTTAAGAATAAAAACACTAGCAGTGAGTTAGGACATCT
AATGCTTTTAAAGATAGATGTATATGTCGCAACGATAGTGTAAGAATCAGCTGAAGCTACCGGTGAGGTTTT
GGTTATTTTCTTTCAGTCATCTGAGTCCATTTCCAATAAAGGTTGTTTTTTTGTTTCGTTTTTTGTTTTTTTT
TTAATGGGGACAATGAATGAGACTTGTAATATGCAGCAGGAAGAAGTTTCACCAAGACTTTGAAACTGGCAA
GAATGATCCAGATGTCCAATTGCCTCTACTTAACCTTATTCGTCTACCTTCAGAAGTAATTAGATTCTCTTC
CACACTAATTCGTATCCCCCATCGCTCTGCTTTTCACCCACTACGTTGTTCCAAGATTCATCCTCAGGGGAA
AAAATTGAGAGCCACACTTTCGGCTCCACCTGCAGCTCCTGGAAATCAAATGGTGAAGTTATAAGAAGTACC
TTAACTTGGAGTGAAGTGGCCTAGTCATAAGTTTTGCCCTATTCCTTGCCAGGGGGTGGGGCGGGGCTTCCT
TCAGTCATCCAGTCTTTATTCACCCGCAGGTACCCCCAGATCGATCCCCCT(**TCATTGACTCCGAGGGGATA
CAGCAGCAATTCATGTTTTGAAGTGTTCTAAATGGTTCAAAACGTGAGGCGCTGCTATACCCCCTCGTGGGG
AAGGTAGAAGGTGGGGTCT) miR424 sequence**

We obtained the human miR-424 promoter and predicted FOXO1 binding sites using the JASPAR database (http://jaspar.genereg.net/cgi-bin/jaspar_db.pl?rm=browse&db=core&tax_group=vertebrates). Accordingly, three primers were designed and then PCRs were performed for the analysis of

FOXO1 binding to the promoter of miR-424. The oligonucleotides were as follows:

The primer at -1559bp that encompass FOXO1 BS1 of the human miR-424 promoter(5’to 3’):

Forward: CCTTTGACACCGATGCTGTT

Reverse: TCCCAACATTTTGTTCCACA

MA0480.1	Foxo1	9.284	0.883446088820745	679	689	1	TTTTGTTTTTC

The primer at -1418bp that encompass FOXO1 BS2 of the human miR-424 promoter(5’to 3’):

Forward: TGTTGGGAGAAAGTTGTGGA

Reverse: CCATCTTGTTTTGGCAATGA

MA0480.1	Foxo1	7.195	0.851567806040198	821	831	1	ACATGTTTTTT

The primer at -412～-443bp that encompass FOXO1 BS3 of the human miR-424 promoter

(5’to 3’) :

Forward: AAGCTACCGGTGAGGTTTTG

Reverse: TGAAACTTCTTCCTGCTGCAT

MA0480.1	Foxo1	9.477	0.886391282012054	1801	1811	1	GGTTGTTTTTT
MA0480.1	Foxo1	9.710	0.889946877937314	1821	1831	1	TTTTGTTTTTT

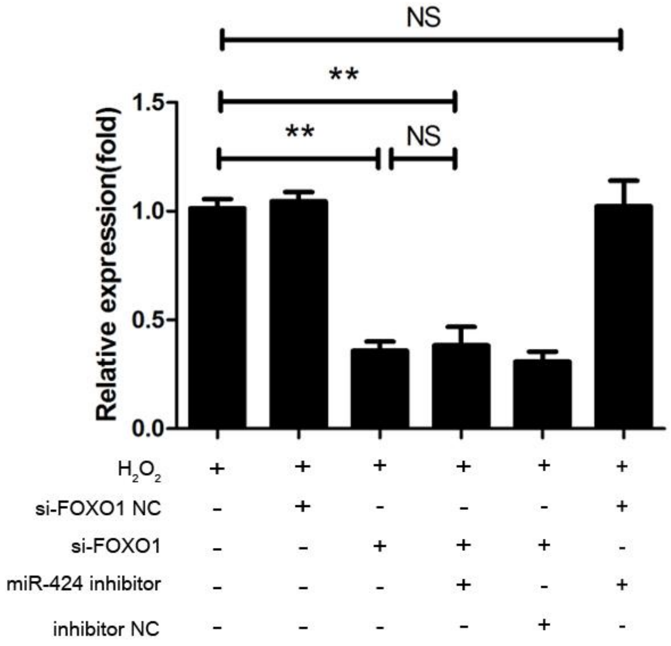

Supplementary Figure 1: FOXO1 mRNA levels after the knockdown of FOXO1 alone and the concomitant knockdown of FOXO1 and miR-424
